# External Cavity Quantum Cascade Laser Vibrational Circular Dichroism Spectroscopy for Fast and Sensitive Analysis of Proteins at Low Concentrations

**DOI:** 10.1021/acs.analchem.4c03498

**Published:** 2024-11-22

**Authors:** Daniel-Ralph Hermann, Georg Ramer, Bernhard Lendl

**Affiliations:** ^†^Research Division of Environmental Analytics, Process Analytics and Sensors, Institute of Chemical Technologies and Analytics; ^‡^Christian Doppler Laboratory for Advanced Mid-Infrared Laser Spectroscopy in (Bio-)Process Analytics, TU Wien, Getreidemarkt 9, 1060 Vienna, Austria

## Abstract

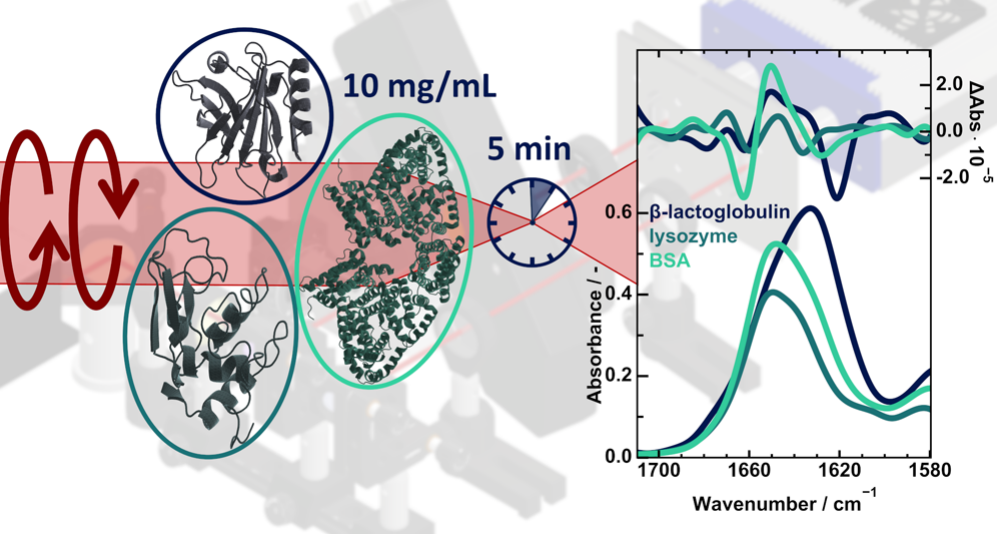

Proteins are characterized by their complex levels of structures, which in turn define their function. Understanding and evaluating these structures is therefore crucial to illuminating biological processes. One of the possible analytical methods is vibrational circular dichroism (VCD), which expands the structural sensitivity of classical infrared (IR) absorbance spectroscopy by the chiral sensitivity of circular dichroism. While this technique is powerful, it is plagued by low signal intensities and long measurement times. Here we present an optical setup leveraging the high brilliance of a quantum cascade laser to measure proteins in D_2_O at a path length of 204 μm. It was compared to classical Fourier-transform infrared spectroscopy (FT-IR) in terms of noise levels and in its applicability to secondary structure elucidation of proteins. Protein concentrations as low as 2 mg/mL were accessible by the laser-based system at a measurement time of 1 h. Further increase of the time resolution was possible by adapting the emission to cover only the amide I’ band. This allowed for the collection of spectral data at a measurement time of 5 min without a loss of performance. With this high time resolution, we are confident that dynamic processes of protein can now be monitored by VCD, increasing our understanding of these reactions.

Asymmetric structures are a common occurrence across the chemical world, being present in small molecules up to large biological systems.^1,2^ In the context of chemistry, asymmetry is part of the broader context of chirality, defined as the inability of a structure to be superimposed on its mirror image.^3^ For small molecules, this quality results in the presence of enantiomers or diastereomers, depending on a change in rotation in one or more chiral centers, respectively.^4^ This quality also translates to biological systems, as e.g., proteinogenic amino acids in most organisms are made up exclusively of l-amino acids.^5^ In addition to this fundamental level, the macromolecules formed by biopolymers, e.g., proteins or nucleic acids, also present a form of chirality.^6,7^ Based on the different side chains, the amino acids backbone arranges itself into either α-helices, β-sheets, random coils and turn structures.^8^ These secondary structures constitute the basis for the subsequent orientation into tertiary or even quaternary structures, which again define the function of the protein. Proteins operate for the most part either as biocatalysts, e.g., enzymes, or as receptors. Both types of reactions rely heavily on geometric recognition of the target molecule by the biopolymer, warranting a closer look on both structures.^7^

Consequently, both the analytics of chirality and specifically of protein structure are the target of intensive research.^9,10^ Since proteins are mostly involved in chemical reactions, there is a need for analytics able to operate at high time resolution and with proteins in their natural environment, i.e., aqueous solutions.^11,12^ Vibrational spectroscopy, specifically infrared (IR) absorption spectroscopy satisfies this criteria and has been used to study proteins for decades now.^13,14^

IR spectroscopy relies on vibrational and rotational movements of molecules under excitation with light between 2.5–25 μm, commonly collected by a Fourier transform infrared spectrometer (FT-IR).^15^ Protein studies are based on vibrations occurring in their peptide backbone, with the most intense bands being the amide I and amide II bands.^16^ The amide I band (1700–1600 cm^–1^) mostly originates from the C=O stretching vibration and is predominantly used for structure assignment. In contrast, the amide II band (1600–1500 cm^–1^) is a combination band of the N–H in plane bending and the C–N stretching vibrational modes.^16^ The intensities, band shapes and peak maxima of these peaks change drastically as a consequence of the dipole–dipole interactions and hydrogen bonding arising from different secondary structure orientations. Therefore, the structure of the proteins can be assessed based on these characteristics, with increasing predication accuracy achieved by chemometric evaluation (partial-least-squares, multivariate curve resolutions-alternating least-squares or band fitting).^8,17−19^

If more in depth information is necessary, the method of classical IR absorption spectroscopy can be augmented by including a polarization modulation scheme.^20,21^ By generating alternately left and right handed circularly polarized light and calculating the difference in absorbance between them vibrational circular dichroism (VCD) can be measured. Chiral structures exhibit substantially different VCD signals, e.g., enantiomers lead to bands with opposite signs. VCD is therefore capable of determining the absolute configuration of small molecules in solution, leading to its broad usage in the pharmaceutical industry.^22,23^ While such a clear assignment is of course impossible for proteins, the added chiral dimension can improve the prediction accuracy for secondary structure determination.^6,12^

Protein VCD signals are located in the same region as their IR absorbance counterparts, but are characterized by sharper bands, and the occurrence of both positive and negative peaks. Unfortunately, VCD signals also differ from their parent absorbance band by a decrease in intensity of ∼10^–4^, necessitating low noise and therefore time-consuming spectral acquisitions up to 12 h. In an effort to keep the routine measurement time down to a few hours, VCD spectra of biomolecules are generally collected at a spectral resolution of 8 cm^–1^, allowing for faster scan acquisition times in an FT-IR instrument.^24−26^

For protein structure analytics the low signal intensities of VCD are exacerbated by the interfering absorption of the HOH-bending vibration of water at ∼1643 cm^–1^.^16,17^ The resulting decrease in spectral throughput limits the useable optical path length for classical FT-IR spectroscopy to <10 μm and for FT-IR VCD studies to ∼6 μm, with correspondingly high concentrations >100 mg/mL for VCD measurements.^17,25,26^ This can lead to problems with solubility and crowding effects, making VCD protein studies challenging. One way around this problem is to replace H_2_O by D_2_O, since the higher mass of deuterium shifts the bending vibration to 1200 cm^–1^, leaving the amide bands unobstructed.^10^ Consequently pathlengths between 25 and 50 μm can be routinely used for protein VCD studies in D_2_O.^26−29^

Besides changing the experimental conditions, the use of a more intense light source can offset the high absorbance of H_2_O. In the mid-IR range, this became possible with the development of quantum cascade lasers (QCL). By relying on inter sub band transitions as opposed to the interband transitions underlying classical laser designs, QCLs emit high power, highly polarized light tunable over the infrared spectral region.^30,31^ Modern external cavity QCLs (EC-QCL) incorporate a grating, allowing for a coverage of up to 500 cm^–1^ by a single chip laser. This broad coverage also makes protein IR studies possible, with the high power of the laser facilitating the use of higher pathlengths. Indeed, laser-based IR spectrometers were employed for protein structure analytics in H_2_O (∼25 μm path length) and D_2_O (∼478 μm) at comparatively low concentrations.^10,11,17^

The use of QCLs for VCD spectral acquisition was first reported in 2011 for small molecules in CDCl_3_ and H_2_O between 1320 and 1220 cm^–1^.^32^ However, QCL-VCD remained a niche application and failed to outperform FT-IR VCD spectrometer in terms of signal-to-noise levels. Beginning with 2020, renewed interest led to a number of publications on the subject from multiple groups.^33−36^ These outlined more evolved optical design, e.g., QCL-VCD microscopy, and also contained some studies on peptides in H_2_O and D_2_O at 25 μm, although with no accessible amide I VCD vibration. Furthermore, it was finally possible to outperform FT-IR VCD in terms of signal-to-noise ratio, by means of balanced detection. This scheme is used to compensate for the pulse-to-pulse fluctuations and 1/*f* noise originating in the laser.^35,37^

Building upon the recent advancements, we present a balanced detection QCL based instrument for VCD measurements of low concentrated proteins in D_2_O. The acquired spectra are compared in terms of noise and band position with reference FT-IR VCD spectra. The accessible concentration range for the 204 μm path length cell is evaluated and proteins comprised of different secondary structures are compared. Additionally. further possible improvements of time resolution and sensitivity are discussed.

## Experimental Section

### Instrumental Setup

The reference FT-IR VCD and absorbance spectra were collected with a Vertex 70v spectrometer equipped with a PMA50 accessory (both Bruker, Germany), containing a 42 kHz photoelastic modulator (PEM, Hinds Instruments), set to a phase shift of 0.5π at 1555 cm^–1^. A low-pass filter (cutoff: 1828 cm^–1^) was placed before the linear polarizer and a resolution of 8 cm^–1^ was used for both absorbance and VCD spectra. The samples were placed in a 23 μm path length cell with CaF_2_ windows and spectra were collected for 1 h.

**Figure 1:**
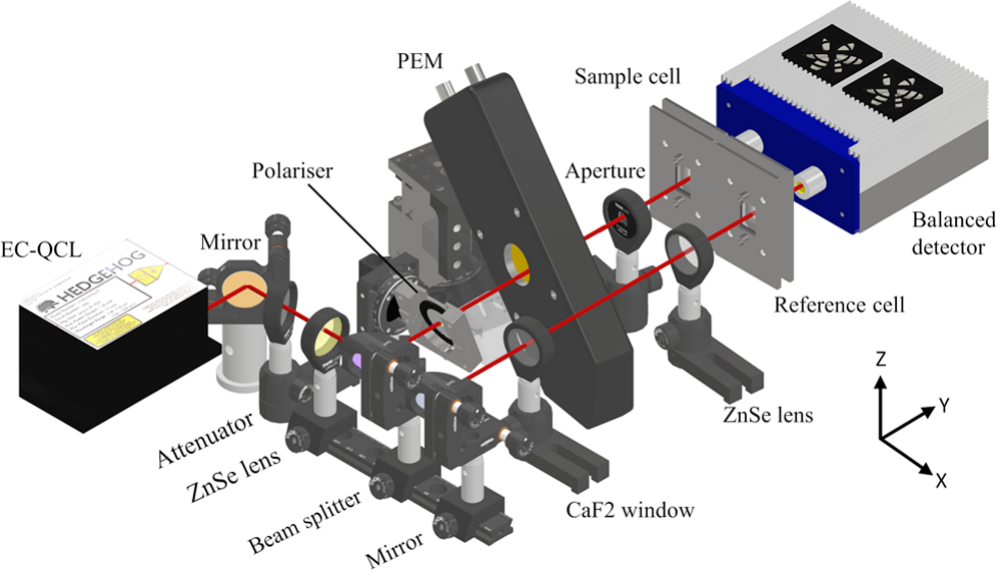
Optical setup used for the QCL-VCD measurements of proteins in D_2_O.

The laser-based instrument used for this study is an optical setup developed specifically for low noise QCL-VCD measurements. It is based on an iteration of our previously published balanced detection system and can be seen in Figure 1.^35^

An EC-QCL (Daylight Solutions Inc.) tunable between 1360 and 1760 cm^–1^ and operated at 860 mA provided the high-power IR light used for the measurements. It was operated at 400 kHz pulse repetition rate with a pulse duration of 700 ns and set to a temperature of 19 °C. Excess heat was removed by a liquid cooling system. Following redirection by a gold mirror, the laser light was attenuated by a reflective attenuator (gold sputtered CaF_2_ window). This attenuation was necessary in order to keep the laser intensity in the linear range of the detector. An attenuation of the laser intensity by reducing the laser current was not a valid alternative, as this would have resulted in a limited spectral range.

Subsequently, the laser beam was focused on the sample cell by means of a 200 mm ZnSe lens (AR coated, Thorlabs Inc.). After the lens, the beam was directed onto a CaF_2_ beam splitter (Thorlabs Inc.), with the reflected and transmitted beam designated the sample and reference beam, respectively. The transmitted beam was directed through an angled CaF_2_ window, acting as an attenuator, and refocused by a 50 mm ZnSe lens. This was done to keep the intensity reaching the reference detector as equal as possible to the one for the sample detector over the whole tuning range.

The sample beam on the other hand first passed a KRS-5 wire grid polarizer (1:300 extinction, Optometrics), used to improve the polarization purity of the laser emission. This was necessary since closer inspection of the laser’s polarization, see Figure S1, revealed an elliptic polarization with vertical orientation. The polarizer was tilted around the *X*-axis by 35°, reducing the reflection for the vertically oriented laser emission according to the Fresnel equations. This was done to reduce interference effects arising from the coherent laser beam. Subsequently a PEM was placed in the sample beam. This optical element modulated the polarization at a frequency of 50 kHz and was set to 0.5π at 1555 cm^–1^. The PEM was tilted by 10° around the *Z*-axis, to shift the reflected beam from the transmitted one. The beam diameter reduction by the ZnSe lens enabled the complete separation of the beams, with the reflected beam being blocked by a 1 mm aperture (Thorlabs Inc.).

After passing the sample and reference cell, both at 204 μm, the respective beams were collected by the balanced detection module (VIGO Photonics S.A., Poland). The detector elements were thermoelectrically cooled to 201 K and closely matched in their detectivity. The optical setup was built on a temperature stabilized breadboard (300 mm × 450 mm, Thorlabs Inc.) set to 22 °C, which approximately corresponded to room temperature. Furthermore, it was enclosed in an acrylic glass housing and flushed with dry air to prevent water vapor interference.

### Data Acquisition

To extract the correct intensities corresponding to the laser and VCD channel respectively phase sensitive detection was implemented by a MFLI lock-in amplifier (with the F5M and MD extensions, Zurich instruments, Switzerland). The reference signal from the PEM controller was fed to the trigger input of the MFLI and one oscillator was set to the eighth harmonic (400 kHz) of this signal, with the corresponding demodulator outputting a reference signal at this frequency. This signal was used to time the laser pulsing scheme in reference to the PEM modulation cycle, enabling the utilization of 25% of the laser intensity at the maximum of the PEM cycle, see Figure S2. The high duty cycle of 28% ensured efficient detection by the lock-in amplifier. A scan trigger connected the laser controller to the MFLI, enabling the referencing of the data acquisition to the spectral sweep.

During the spectral acquisition, the balanced detector signal was collected. This signal is the result of the subtraction between the sample and reference detector, providing the noise reduction characteristic for balanced detection. The collected signal was demodulated at the PEM’s fundamental frequency  and its eighth harmonic . Once before the measurement, the reference detector signal  is collected to provide an offset for the balanced detector signal. The VCD signal as a function of the wavenumber (ν ®) can then be calculated according to
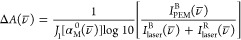
1with  being the first order Bessel function, its argument is the amplitude of the phase shift applied by the PEM .

For this study, the laser was continuously sweeping between 1400–1710 or 1580–1710 cm^–1^ at a speed of 40 cm^–1^/s, and the signal was collected at 838 Sa/s (∼20 samples/cm^–1^). For each covered area, the number of sweeps per scan were set to result in 5 min of acquisition time.

### Data Evaluation

Both the FT-IR and QCL-VCD scans were baseline corrected to compensate for drifts before averaging.^38^ The QCL-VCD spectra were collected at an unfiltered resolution of 0.5 cm^–1^ and were smoothed by fitting a third order spline to match the 8 cm^–1^ resolution of the FT-IR spectra.^39^ Both the absorbance and VCD spectra were corrected by the D_2_O background.

### Chemicals and Sample Preparation

Bovine serum albumin (BSA, purity ≥98%), lysozyme from hen egg white, β-lactoglobulin from bovine milk (purity ≥90%) and D_2_O (99.9% D) were purchased from Sigma-Aldrich and used as received. The analytes were dissolved in the appropriate volume of D_2_O and used in a timely manner. For the comparison spectra, the concentration for FT-IR VCD was prepared and an aliquot was diluted for the QCL-VCD measurements. For the BSA calibration curve, a stock solution of 48 mg/mL was diluted according to the desired concentration. Around 100 and 300 μL of the prepared solutions were used to fill the FT-IR and QCL cells, respectively.

## Results and Discussion

### Noise Comparison

**Figure 2:**
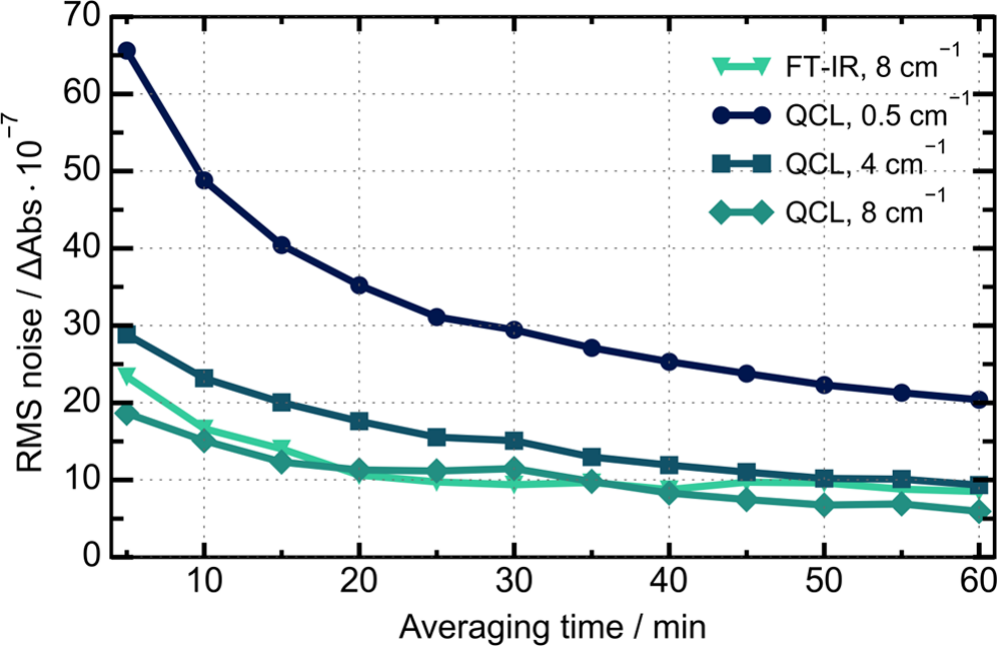
Root mean square (RMS) noise as a function of measurement time for the FT-IR VCD spectra and the QCL-VCD spectra at different smoothing settings. The data was calculated for the area between 1405 and 1705 cm^–1^.

Before actual protein VCD measurements the noise floor for the QCL setup had to be evaluated. For this purpose, 24 D_2_O scans at 5 min each were collected for each system and the data set was split in half to generate a background and a sample block. The difference between the background and sample spectra was calculated for an increasing number of averages. The resulting root-mean-square error (RMS) against the measurement time can be seen in Figure 2, plotted at the native QCL resolution and at different smoothing settings. At the native QCL resolution of 0.5 cm^–1^, the spectra are quite noisy due to an overlaying interference pattern originating in the wire grid polarizer, see also Figure S3.

Subsequent smoothing to a resolution of 4 and 8 cm^–1^ respectively removed the distortions and the noise floor approached the FT-IR level. Indeed, at the maximum averaging time of 60 min the noise levels are at the same level, with a slight advantage for the QCL when compared to the FT-IR at the same resolution. Therefore, we could assume that the spectra collected on both instruments are of comparable quality. Additionally, the higher path length used during the EC-QCL VCD measurements (204 μm vs 23 μm) should translate to a higher signal-to-noise (SNR) by a factor of approximately 9. Consequently, a more encompassing comparison between FT-IR VCD and QCL-VCD for protein studies could be performed.

To evaluate the resolving power of the system in terms of secondary structure determination, three proteins were selected for measurement. Bovine serum albumin is composed mainly of α-helices, with no β-sheet contributions. Lysozyme in contrast is a combination of α-helices and turn structures, with a small fraction of β-sheet. The last protein studied is β-lactoglobulin, a milk protein composed of a mixture of α-helices and β-sheets^9,20^

**Figure 3:**
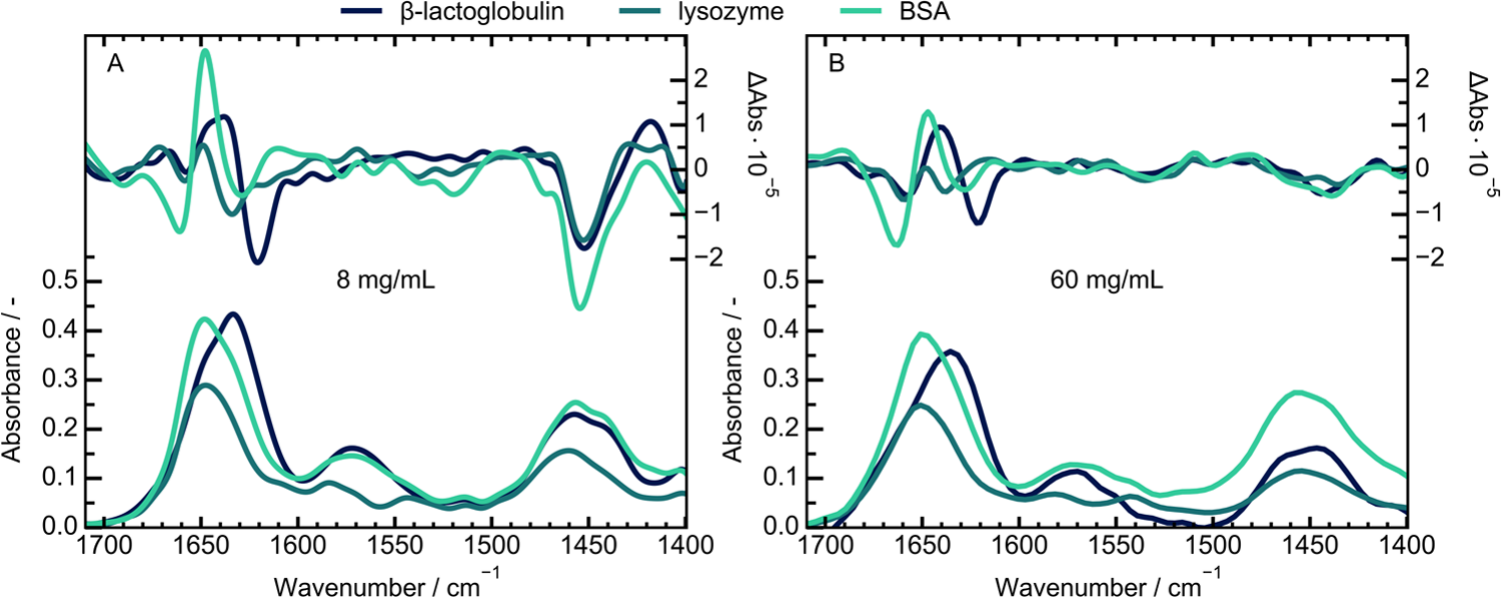
Absorbance and VCD spectra of β-lactoglobulin, lysozyme and bovine serum albumin (BSA) in D_2_O. The spectra were collected with the QCL-VCD setup (A) at a concentration of 8 mg/mL and a commercial FT-IR VCD spectrometer (B) at a concentration of 60 mg/mL.

For the FT-IR samples, a concentration of 60 mg/mL was chosen, to ensure an adequate solubility for all protein samples. The QCL samples were diluted to 8 mg/mL to ensure a better comparison, since with the higher path length of this system a concentration of 60 mg/mL would lead to total absorption. The spectra for both systems were collected for 1 h and are depicted in Figure 3A,B for the QCL and FT-IR system, respectively. For better comparison, a direct overlay of the respective protein spectra collected at the different instruments can be found in Figure S4.

When comparing the classical absorbance spectra between the proteins, there are some differences for the amide bands. Due to the vibrations, it is based on, the amide II band also shifts alongside the H_2_O–D_2_O shift. Consequently, the amide II area is less useful for evaluation, since it is influenced by the degree of deuteriation achieved for the proteins. In addition, due to the hygroscopic nature of D_2_O a DOH-bending vibration can occur at ∼1455 cm^–1^, which distorts this spectral region.^40^ This is most probable the origin of the shift in intensity at the amide II’ region which translates also to the VCD spectra. We will therefore focus on the amide I’ band for the purpose of this study.

For the amide I’ band of both BSA and lysozyme the band maximum lies at ∼1649 cm^–1^, with the only difference being a difference in intensity. These characteristics are present in both systems used and also agree well with literature values.^9,26^ In contrast, the β-lactoglobulin band maxima is shifted to ∼1636 cm^–1^,with a slight shoulder at ∼1652 cm^–1^, which again agrees well with literature.^11,41,42^ So, while the spectra agree well with expectations, a difference according to secondary structure components cannot be easily assigned, at least between lysozyme and BSA.

This is different when the focus is switched to the corresponding amide I’ VCD bands. For BSA, a strong couplet between 1662 and 1648 cm^–1^ can be found in both systems. In addition, the deuteriation of the protein leads to a sideband at 1628 cm^–1^.^20^ A difference between the systems can be observed for the relative height of the positive band, which we attribute to shifts arising from the baseline correction and calibration.

As for the lysozyme band shape, it exhibits two negative peaks at 1660 and 1635 cm^–1^. Again, the difference in intensity varies between the systems, which can be attributed to the sensitivity of lysozyme to incomplete deuteriation.^26^ In contrast, the other studied proteins are not affected by deuteriation in a similar way and their bandshapes are stable.^21,43^

The amide I’ band of β-lactoglobulin contains a characteristic set of peaks, with one small negative band at 1660 cm^–1^, a positive band at 1638 cm^–1^ and one strong negative band at 1621 cm^–1^. These bands agree well between the systems as well as with literature values.^44^

For the sample set of proteins, the different combinations of secondary structures led to characteristic peaks in the VCD amide I’ bands. Specifically, the strong positive couplet of BSA is indicative of a high α-helical structure, while the negative couplet with an additional sideband of β-lactoglobulin is an indicative of its β-sheet content. The w-shape seen for lysozyme is typical for a protein composed of mixed secondary structure combinations.^9,21,43,45^ In contrast, the assignment based on amide I’ absorbance data was less conclusive. This is also true for the QCL-VCD spectra, which matches the FT-IR reference spectra well and allows for the evaluation of protein VCD bands at a concentration of 8 mg/mL within 1 h of measurement time.

### Accessible Concentration Range

**Figure 4:**
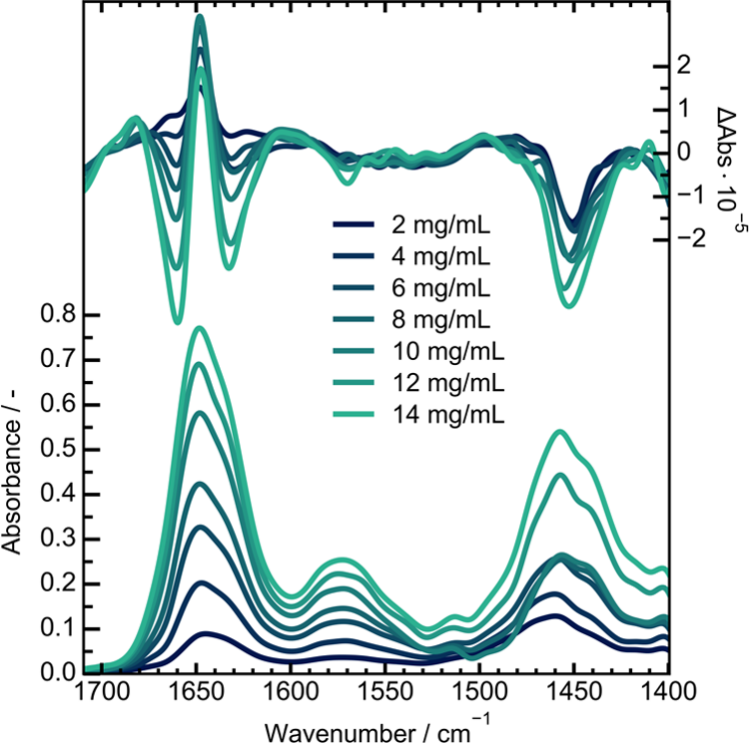
VCD and IR absorbance spectra collected for BSA in D_2_O in varying concentrations.

With the validity of QCL-VCD protein spectra confirmed against FT-IR reference measurements, the sensitivity of the system needs to be evaluated. For this purpose, a dilution series of BSA in D_2_O was prepared and the measurement time was again 1 h. The concentrations ranged from 2–14 mg/mL, corresponding to maximum absorbance values between 0.08 and 0.78. Figure 4 depicts the resulting VCD spectra and their corresponding absorbance spectra. The intensity of the amide I’ absorbance and VCD band follows a linear relationship against the concentration even down to 2 mg/mL. With the resulting linear fit, see Figure S5, the limit of detection (LOD), corresponding to a SNR of 3, could be estimated. For the noise (5.93 × 10^–7^ ΔAU) at the used 1 h acquisition time, this results in a LOD of 0.32 mg/mL. An improvement of this value is possible by increasing the number of averaged spectra, reducing the noise in the process. Alternatively, the signal could be increased by increasing the path length of the transmission cell. This constitutes a promising approach, as for classical IR absorbance studies pathlengths of up to 478 μm have been reported.^10^ However, this was not feasible for this study, as D_2_O shows a small absorption feature ∼1550 cm^–1^, which is compounded to a significant decrease of the laser intensity over a broad area, see Figure S6. Since the maxima of the laser spectral emission profile lie outside of this area, this high absorbance could not be adequately compensated.

Even so, the shown performance benefits quite substantially from the longer path length available for QCL-VCD studies. Comparatively low concentrated samples could be measured and their VCD bands were well resolved (Figure 4).

**Figure 5:**
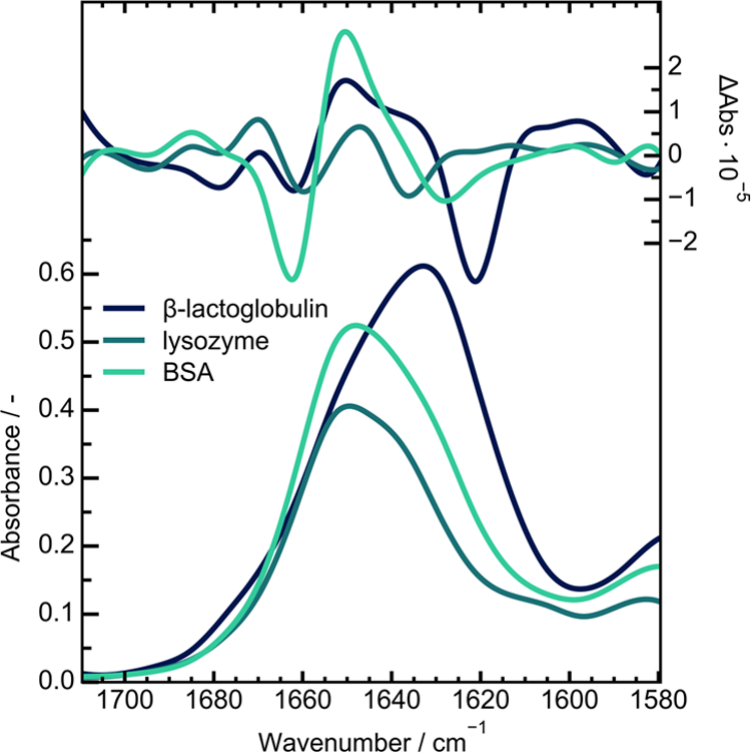
IR absorbance and VCD spectra of 10 mg/mL of β-lactoglobulin, lysozyme and BSA in D_2_O. The spectra were acquired between 1710 and 1580 cm^–1^ after a measurement time of 5 min.

### Further Improvements of Acquisition Speeds

With the performance of the system for broadband spectral acquisition of proteins in D_2_O established, the possibility of even faster acquisition speeds was explored. EC-QCL instruments scan over their accessible wavelength range by changing the angle of an internal grating. Therefore, the spectral information is gathered as a function of time and the measurement time is directly proportional to the spectral coverage. One can make use of this characteristic if only a few spectral features contain the necessary information, e.g., protein studies. Since most studies of secondary structure evaluation base their analysis on the amide I’ absorbance and VCD bands, data containing only these bands should be sufficient.^10,18,20,26^

Following this logic, we let the laser sweep between 1710–1580 cm^–1^, covering the amide I’ band and some additional baseline on both sides. This enabled the collection of more spectra per unit of time, reducing the noise as consequence of the increased number of averaged scans. A comparison of the noise levels for D_2_O spectra, see Figure S7, confirmed this expectation. Noise level improvements were observed down to 2.73 × 10^–7^ ΔAU for the tested maximum acquisition time of 30 min. However, even for 5 min of spectral averaging the noise level was deemed sufficient for protein measurements.

For these measurements, 10 mg/mL solutions each of the studied proteins were prepared. This concentration was chosen to better represent a typical VCD experiment, where the desired absorbance lies between 0.4 and 0.8 AU. Figure 5 depicts the collected IR and VCD measurements for 5 min of averaging. The spectral features are well resolved, even for the low intensity signals of lysozyme. The influence of the secondary structure on the corresponding VCD bands is again visible and corresponds well to the broadband data. Accordingly, it can be said that protein studies in D_2_O can be performed at 5 min of measurement time at adequate noise levels, if the information is contained in the amide I’ band.

## Conclusions

In conclusion, EC-QCL based analysis of proteins in D_2_O enabled the use of a comparatively long path length, making lower concentrations of proteins accessible to evaluation by VCD. The achieved noise for the spectral range between 1400–1710 cm^–1^ was comparable to the noise floor of FT-IR for the same measurement time. The increased analyte signal due to the longer path length allowed for LOD values of 0.32 mg/mL at measurement times of 1 h. At this measurement time, it was also possible to discriminate the influence of different secondary structure composition on the VCD bands.

Further reduction of the measurement time was made possible by adjusting the laser to only cover the spectral range between 1580–1710 cm^–1^. With this mode of operation, the amide I’ VCD band of different proteins could be resolved even at 5 min of acquisition time. This opens up applications of VCD to support classical absorbance studies like thermal stability investigations by providing additional chiral information at similar time scales.^10,42^

For the sake of completeness, it has to be said that the advantages of QCLs demonstrated here can be most efficiently leveraged when a small and well-defined spectral area is of interest, e.g., amide bands of proteins. For more complicated sample-matrix systems or more in-depth studies the broad coverage offered by FT-IR VCD instruments still has the upper hand in the foreseeable future. Furthermore, a chip with a maximum emission near 1555 cm^–1^ would increase the spectral throughout, allowing for an even longer path length and increased sensitivity.

Nevertheless, we believe that the system shown here, and the data generated is proof of the utility of EC-QCL VCD for biomolecules. Especially for protein studies, oftentimes only a small spectral area carries sufficient information, and high sensitivity and high time resolution are more important than broad applicability. This is specifically the advantage our EC-QCL VCD system enjoys over commercial instruments. We believe this can be leveraged to provide additional information compared to classical IR absorbance evaluation of proteins.

## Data Availability

The experimental data and corresponding data evaluation is available on Zenodo in the form of a Docker container (10.5281/zenodo.12666142).

## Supporting Information Available

Stokes parameters of the laser source (Figure S1); digitized detector signal (Figure S2); unfiltered baseline (Figure S3); overlay of the QCL and FT-IR spectra (Figure S4); calibration lines for BSA in D_2_O (Figure S5); D_2_O absorbance and laser emission spectrum (Figure S6), and RMS noise against measurement time (Figure S7) (PDF)
