# Supplementary material for: External Cavity Quantum Cascade Laser Vibrational Circular Dichroism Spectroscopy for Fast and Sensitive Analysis of Proteins at Low Concentrations
Source: Anal Chem. 2024 Nov 22;96(49):19363–9. doi: 10.1021/acs.analchem.4c03498 (PMC11635754; doi:10.1021/acs.analchem.4c03498)
Supplement: Supplementary file 1 — ac4c03498_si_001.pdf [file ac4c03498_si_001.pdf]

**Electronic supplementary information for:**

# External cavity quantum cascade laser vibrational circular dichroism spectroscopy for fast and sensitive analysis of proteins at low concentrations

**Daniel-Ralph Hermann, Georg Ramer and Bernhard Lendl**

## Content:

Figure S 1. Stokes parameter and degree of polarisation (DOP) collected for the used EC-QCL as a function of wavenumber. The edges of the spectral range are not shown due to artifacts arising from the low laser emission in these regions. The interference fringes at ~ 10 cm-1 spacing originate from the quarterwaveplate used for the collection of the data. ........ 2

Figure S 2. Digitized detector signal as a function of time, with the phaseshift applied by the PEM overlaid. A polarizer oriented horizontally (crossed in relation to the laser polarisation) was placed behind the PEM and the laser pulsing scheme was set in a way that 2 of 8 laser pulses during the PEM cycle are located at the PEM maxima. ........ 2

Figure S 3. Unfiltered, baseline corrected $D_2O$ spectrum, presented at the native QCL resolution of 0.5 $cm^{-1}$. The overlaying fringe pattern at a frequency of ~1 $cm^{-1}$ has its origin in the used wiregrid polariser. ........ 3

Figure S 4. Comparison of the QCL (full line) and the FT-IR (dotted line) absorbance and VCD spectra for the different measured proteins ........ 3

Figure S 5. Linear fit obtained for the absorbance and VCD spectra of the BSA calibration line. The maximum absorbance value of the amide I' absorption band and the intensity of the couplet between 1608 and 1680cm-1 are taken as y-data for the respective fit, and the $r^2$, the slope (m) and the offset (b). ........ 3

Figure S 6. FT-IR absorbance spectrum of $D_2O$ in the used 23 µm pathlength cell and laser intensity collected by the reference detector after passing through $D_2O$ in the used 204 µm pathlength cell. Of note is the slight background absorbance band at ~ 1550 $cm^{-1}$, which leads to a strong reduction of the laser intensity for the longer pathlength. ........ 4

Figure S 7. RMS noise as a function of measurement time. The noise for different smoothing settings for the reduced spectral bandwidth is compared to the broadband QCL acquisition system. ........ 4

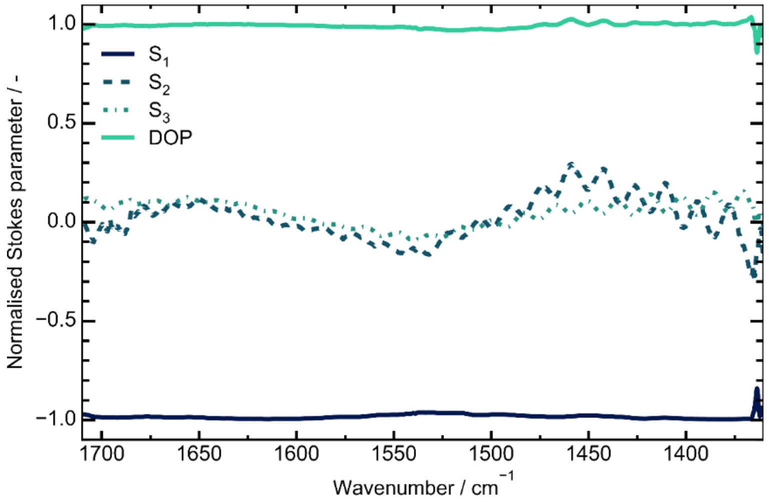


Figure S 1. Stokes parameter and degree of polarisation (DOP) collected for the used EC-QCL as a function of wavenumber. The edges of the spectral range are not shown due to artifacts arising from the low laser emission in these regions. The interference fringes at ~ 10 cm-1 spacing originate from the quarterwaveplate used for the collection of the data.

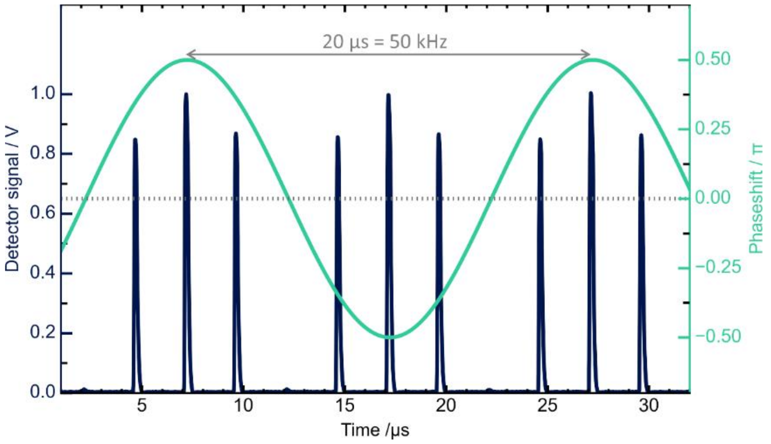


Figure S 2. Digitized detector signal as a function of time, with the phaseshift applied by the PEM overlaid. A polarizer oriented horizontally (crossed in relation to the laser polarisation) was placed behind the PEM and the laser pulsing scheme was set in a way that 2 of 8 laser pulses during the PEM cycle are located at the PEM maxima.

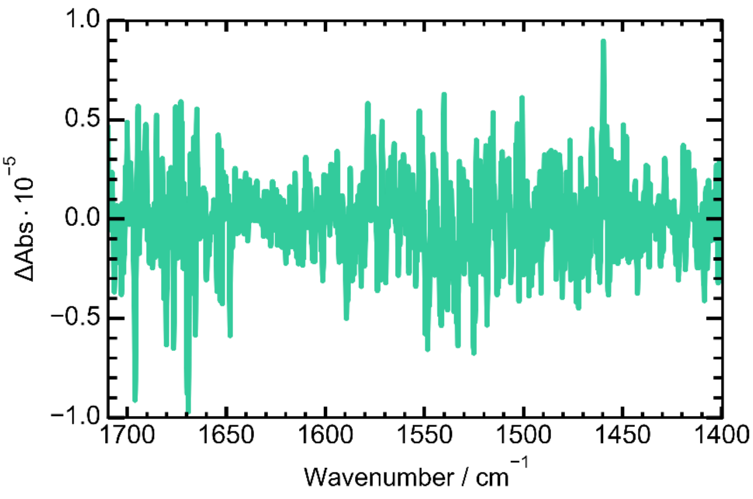


Figure S 3. Unfiltered, baseline corrected $D_2O$ spectrum, presented at the native QCL resolution of 0.5 $cm^{-1}$. The overlaying fringe pattern at a frequency of ~1 $cm^{-1}$ has its origin in the used wiregrid polariser.

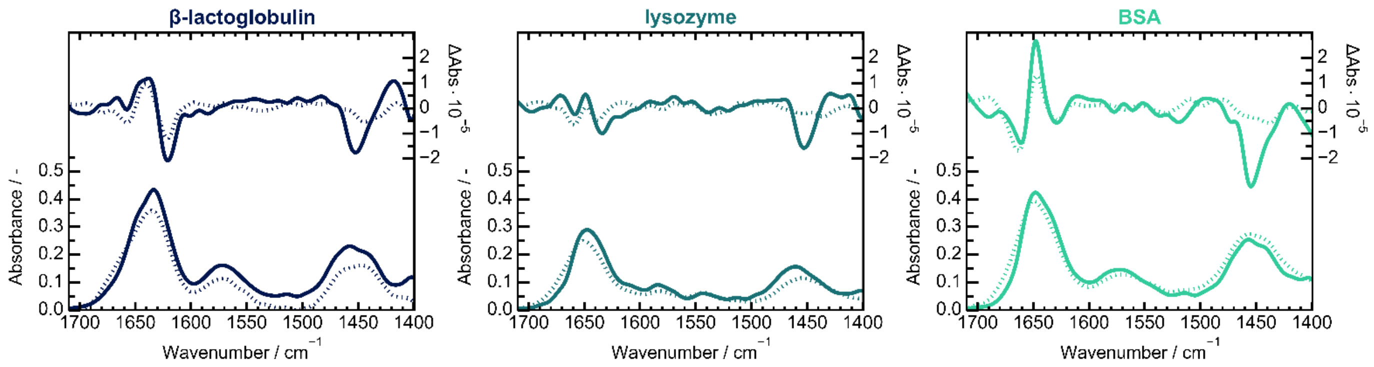


Figure S 4. Comparison of the QCL (full line) and the FT-IR (dotted line) absorbance and VCD spectra for the different measured proteins

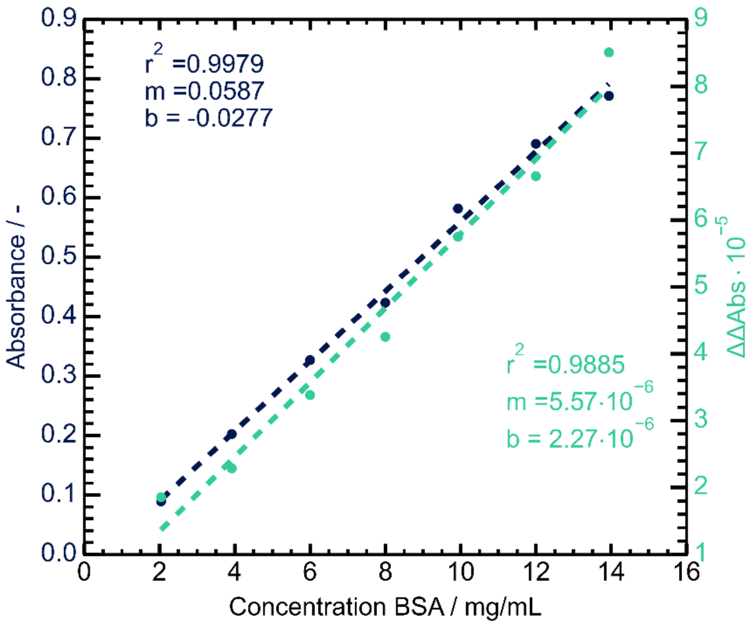


Figure S 5. Linear fit obtained for the absorbance and VCD spectra of the BSA calibration line. The maximum absorbance value of the amide I′ absorption band and the intensity of the couplet between 1608 and 1680cm-1 are taken as y-data for the respective fit, and the $r^2$, the slope (m) and the offset (b).

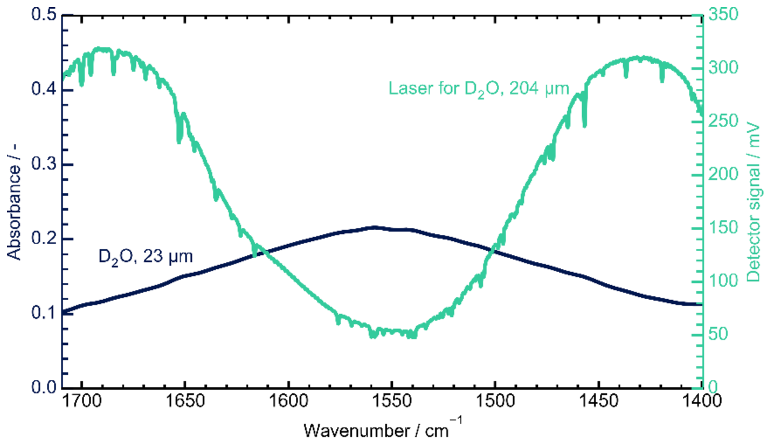


Figure S 6. FT-IR absorbance spectrum of $D_2O$ in the used 23 µm pathlength cell and laser intensity collected by the reference detector after passing through $D_2O$ in the used 204 µm pathlength cell. Of note is the slight background absorbance band at ~ 1550 cm$^{-1}$, which leads to a strong reduction of the laser intensity for the longer pathlength.

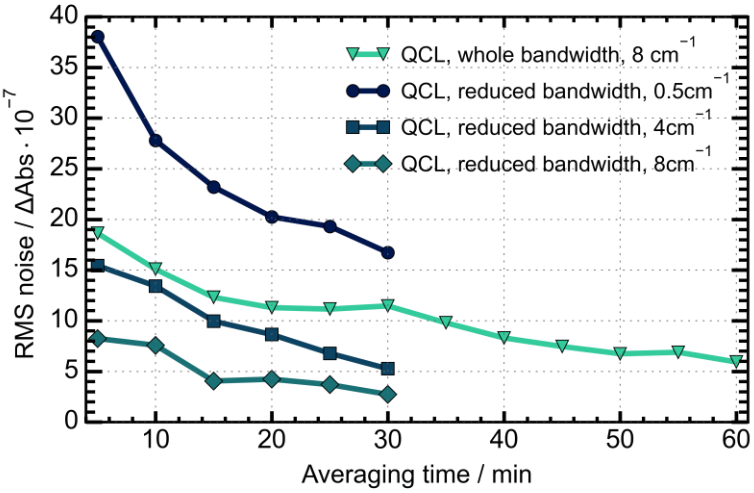


Figure S 7. RMS noise as a function of measurement time. The noise for different smoothing settings for the reduced spectral bandwidth is compared to the broadband QCL acquisition system.
